# P317: Injection safety in health institutions in Benin

**DOI:** 10.1186/2047-2994-2-S1-P317

**Published:** 2013-06-20

**Authors:** S Assavedo, A Attolou Gbohoun, P Soroheye, DP Alahassa, M Amoussou Guenou, B Orou Yorou, KAD Gazard

**Affiliations:** 1 Health Minstry of Benin, Cotonou, Benin

## Introduction

Patient safety is a major global public health challenge each year around the world. The the misuse of injections combines with other harmful practices to cause 8- 16000000 cases of infection by the hepatitis B virus and 80,000 to 160, 000 cases of HIV infection. Among the dangerous practices, reuse of syringes and needles without sterilization is particularly worrying. It becomes imperative to know the current state of injection safety in Benin.

## Objectives

Make on inventory of the situation with regard to injection safety in hospitals in Benin.

## Methods

A descriptive cross-sectional study that targeted providers of public and religious hospitals. A convience sample of are providers present on the day of the survey was assessed. The administration of a questionnaire by personal interview and direct observation techniques were used for data collection.

## Results

**87.4%** of respondents have the necessary hardware for proper injections, **20%** were victims of accidental needle sticks once in the past 12 months. No provider reuses injecting equipment, **82%** meet the standards in their injection practices. **86%** of hospitals eliminate waste in requirements.

## Conclusion

The syringes are not reused hospitals in Benin, waste disposal is provided by post-injection incinerators. The practice of recapping needles persist for some providers. We recommend the development of a national policy on injection safety.

## Disclosure of interest

None declared

